# Importance of the length of the myocardial sleeve in the superior vena cava in patients with atrial fibrillation

**DOI:** 10.1002/joa3.12494

**Published:** 2021-01-07

**Authors:** Eiji Nyuta, Masao Takemoto, Togo Sakai, Takahiro Mito, Akihiro Masumoto, Wataru Todoroki, Keishiro Yagyu, Jiro Ueno, Yoshibumi Antoku, Tokushi Koga, Takafumi Ueno, Takuya Tsuchihashi

**Affiliations:** ^1^ Cardiovascular Center Steel Memorial Yawata Hospital Kitakyusyu Japan; ^2^ Cardiology Munakata Suikokai General Hospital Fukutsu Japan; ^3^ Cardiology Hakujuji Hospital Fukuoka Japan; ^4^ Cardiology Fukuoka Kinen Hospital Fukuoka Japan

**Keywords:** atrial fibrillation, catheter ablation, firing, non‐pulmonary vein foci, superior vena cava

## Abstract

**Background:**

Pulmonary vein (PV) antrum isolation (PVAI) has proven to be a useful strategy for radiofrequency catheter ablation (RFCA) of atrial fibrillation (AF) worldwide. However, non‐PV foci, especially from the superior vena cava (SVC), play an important role in initiating and maintaining AF.

**Methods:**

In all, 427 consecutive patients with non‐valvular AF who were admitted to our hospitals to undergo RFCA of AF using an EnSite^™^ system were evaluated. The length from the top of the sinus node to the top of the myocardial sleeve of SVC (L‐SVC), longer and shorter diameter of SVC of 1 cm above of junction of right atrium and SVC, and local activation time (LAT) of SVC were measured. Then, the SVC firing was evaluated by an intravenous administration of isoproterenol and adenosine triphosphate.

**Results:**

L‐SVC, longer and shorter diameter of SVC, and LAT of SVC were significantly longer in the SVC firing group than non‐SVC firing group (*P* < .05). Moreover, in accordance with the L‐SVC, the frequency of the SVC firing significantly increased (*P* < .001). A univariate analysis and multivariate statistical analysis revealed that L‐SVC longer than 37.0 mm (odds ratio 6.39) and longer diameter of SVC (odds ratio 6.78) were independent risk factors for SVC firing in patients with AF who underwent RFCA of AF.

**Conclusions:**

In view of these findings, L‐SVC longer than 37.0 mm longer diameter SVC longer than 17.0 mm may be one of the important predictors of SVC firing in patients with AF.

## Graphical abstract

An L‐SVC longer than 37.0 mm and longer diameter of SVC longer than 17.0 mm may be one of the important predictors of SVC firing in patients with AF.

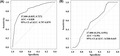

## 1 INTRODUCTION

Pulmonary vein (PV) antrum isolation (PVAI) has proven to be a useful strategy for radiofrequency catheter ablation (RFCA) of atrial fibrillation (AF) worldwide.^1^, ^2^, ^3^ However, non‐PV foci play an important role in initiating and maintaining AF in less than 20% of patients.^4^, ^5^ Non‐PV foci are located at sites including the superior vena cava (SVC), left atrial (LA) posterior wall, crista terminalis, coronary sinus, ligament of Marshall, interatrial septum, left atrial appendage, and so on.^4^, ^5^, ^6^, ^7^, ^8^ In particular, the SVC, which harbors 25%‐40% of the non‐PV foci, is the most common non‐PV origin of AF.^4^, ^9^, ^10^, ^11^ We often experience the atrial myocardium extending into the SVC at RFCA of AF in daily clinical practice. Thus, in this study, we evaluated the relationship between the SVC firing by an intravenous administration of isoproterenol (ISP) and adenosine triphosphate (ATP), which plays an important role in non‐PV foci, and length of the myocardial sleeve in the SVC.

## 2 METHODS

### 2.1 Baseline clinical characteristics

This study was approved by the institutional review committee and ethics review board of our hospitals. From 2018 to 2019, 427 consecutive patients (281 males and 146 females with a mean age of 71.6 ± 9.3 years) with non‐valvular AF who were admitted to our hospitals to undergo RFCA of AF using an EnSite NavX/Velocity™ Cardiac Mapping System (Abbott) were evaluated. The type of AF was determined according to the 2018 JCS/JHRS guidelines on Non‐Pharmacotherapy of Cardiac Arrhythmias.^12^ In brief, paroxysmal, persistent, and long‐lasting AF were defined as AF that occurred from a few minutes to days and then stopped on its own within 7 days, AF that lasted for more than 7 days and did not correct on its own, and AF that consistently exhibited a high, erratic heartbeat that could not be corrected, respectively. The patients with hemodialysis, a history of RFCA with an SVC isolation, chronic obstructive lung disease, which was a contraindication for ATP, who persisted AF after PVAI and cardioversion and/or persistent left superior vena cava, were excluded. All patients had their history recorded, and underwent a physical examination, laboratory analysis, chest radiogram, 12‐lead electrocardiogram, and echocardiography within at least 1 month before admission. The CHADS_2_ score,^12^ chamber size and left ventricular ejection fraction by echocardiography, and anatomy and size of the PVs and LA by computed tomography (Aquilion 64 and Aquilion ONE TSX‐301A; TOSHIBA) were also evaluated before the RFCA.

### 2.2 Procedure for the RFCA of AF

All patients were effectively anticoagulated for at least 1 month before the procedure. The procedures were performed after cardiac CT and/or transesophageal echocardiography to rule out any LA thrombi. All patients gave their informed consent. RFCA was performed as described previously.^13^, ^14^, ^15^ In brief, deep sedation with supraglottic airways (i‐gel™; INTERSURGICAL Ltd.) was performed for the RFCA of AF under an intravenous administration of propofol and dexmedetomidin. The initial i‐gel™ size chosen was based on the manufacturer's recommendation according to the body weight.^16^ A temperature probe (SensiTherm™; St. Jude Medical) for monitoring the esophageal temperature was inserted through a side hole of the i‐gel™ under deep sedation, and placed between the level of the left superior and inferior pulmonary veins for both groups. Femoral arterial access was routinely acquired for continuous blood pressure and heart rate monitoring. A 100 unit per kilogram administration of heparin was administered following the transseptal puncture and heparinized saline was additionally infused to maintain the activated clotting time at 300‐400 seconds. After an Advisor™ HD Grid catheter (Abbott) and circular mapping catheter (Optima™; St. Jude Medical) were positioned in the LA after a double transseptal puncture, the LA was reconstructed by an EnSite™ system using the Advisor™ HD Grid catheter. Then, a circumferential PVAI was performed with an open irrigated ablation catheter (FlexAbility™ or TactiCath SE™; St. Jude Medical) or 28‐mm cryoballoon Arctic Front Advance™ (Medtronic, Inc) under electroanatomic guidance with a 3D mapping system with intravenous administration of ISP. In patients with persistent/long‐lasting AF, when AF persisted after the PVAI, the roof and bottom linear ablation of LA posterior wall was additionally performed, and internal electrical conversion was performed to recover sinus rhythm. The completion of the PVAI was defined as the achievement of bidirectional conduction block between the LA and PVs under the administration of ISP (10‐20 μg/h) and ATP (20 mg bolus iv). Furthermore, if common atrial flutter was previously documented or induced by programmed stimulation after the PVAI, an additional cavo‐tricuspid isthmus line ablation was performed. There was no potential for confounding data between the centers and operators because the procedures for the RFCA of AF were performed by electrophysiologists who were trained in advanced RFCA, and the cases were assisted by two staff members who were well trained in catheterization laboratory practices at our hospitals.

### 2.3 SVC evaluation

**FIGURE 1:**
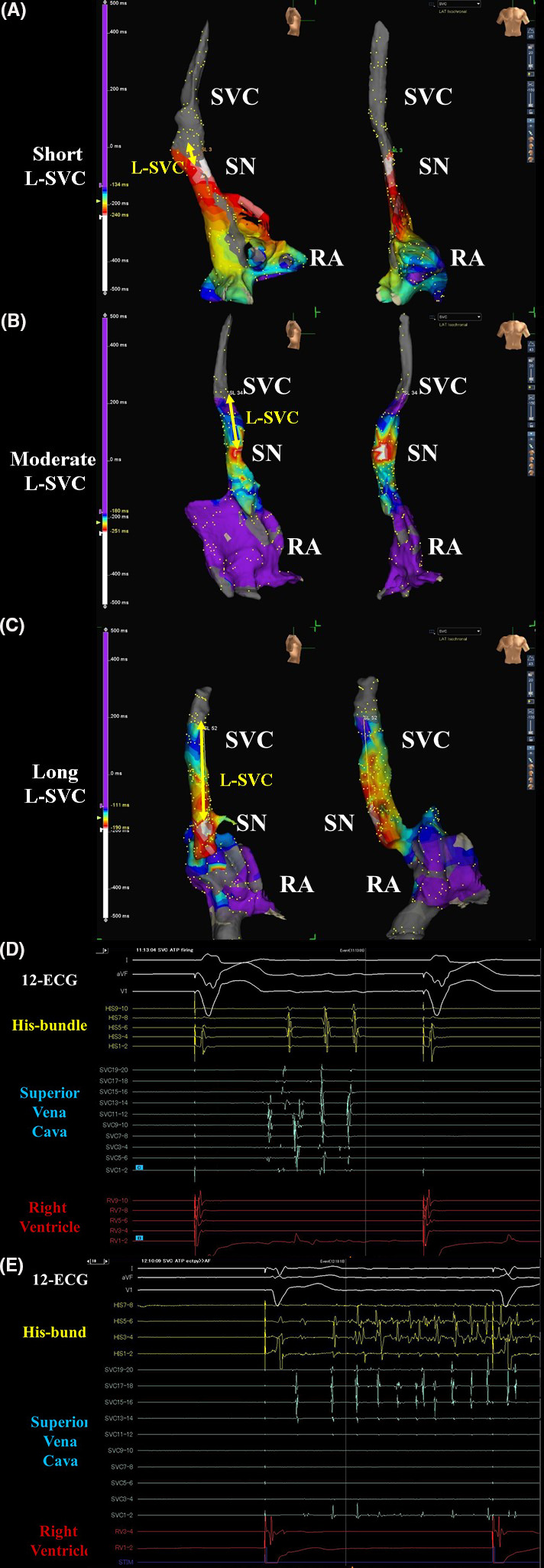
The activation maps of the superior vena cava (SVC) and right atrium (RA), which were reconstructed with EnSite™ 3D‐mapping in the right side (left panels) and frontal (right panels) (A‐C) views. The white areas show the earliest activation sites during sinus rhythm and the white area indicates the sinus node (SN). The length of SVC (L‐SVC) was determined by the length from the top of the sinus node to the top of the myocardial sleeve in the SVC. The intra‐cardiac electrocardiograms show the SVC firing (D) and AF induced by the SVC firing (E) under intravenous administration of isoproterenol and adenosine triphosphate

After the complete PVAI procedure in the LA, the voltage and activation maps of SVC and right atrium were precisely reconstructed by an EnSite™ system using an Advisor™ HD Grid catheter, or circular mapping catheter, Optima™ under an intravenous drip administration of ISP (10‐20 μg/h) during sinus rhythm at a heart rate of 80‐100 bpm (Figure 1A‐C). Therefore, the earliest activation site was the sinus node. The cutoff value of the voltage map was 0.5‐0.1 mV. Less than 0.1 millivolts was defined as no electric potential. Then, the length from top of the sinus node to the top of the myocardial sleeve in the SVC (L‐SVC) was measured. After the circular mapping catheter, Optima™, was placed about 10‐20 mm above the sinus node in the SVC, the SVC firing was evaluated by an intravenous administration of ISP^17^ at 10‐20 μg/h and 30 mg bolus injection of ATP^10^ at least more than twice. If the SVC firing (Figure 1D) and/or AF (Figure 1E) was induced, an SVC isolation was additionally attempted at 45°C with power limit of 20‐25 W for 40‐60 seconds. To prevent diaphragmatic paralysis, just before radiofrequency energy application, we verified that the right diaphragm did not twitch upon pacing from the ablation catheter. The completion of the SVC isolation was defined as the achievement of bidirectional conduction block between the RA and SVC under the administration of ISP and ATP. Additionally, local activation time (LAT) of was measured using an EnSite™ system. Furthermore, the longer and shorter diameter of SVC were evaluated by cardiac CT for RFCA of AF.

### 2.4 Follow‐up post‐ablation

All patients were followed at 1, 2, 3, 6, and 12 months post‐ablation. The ECG was checked periodically, and 24‐hour Holter monitoring was used during first 6 months post‐ablation to assess the recurrence of AF.

### 2.5 Statistical analysis

The numerical results are expressed in the text as the mean ± SD. Paired data were compared by a Fisher's exact test and Student's *t* test or the Wilcoxon signed‐rank test. The trend in the proportions and correlation between the frequency of SVC firing and an L‐SVC was determined by a Cochran‐Armitage analysis.

Comparisons among the three different types of AF were performed using the chi‐square test for categorical variables and one‐way analysis of variance for continuous variables. A multivariate logistic regression analysis was carried out to evaluate the association between the SVC firing and those factors. Factors with at least a borderline significance (*P* < .15) according to the univariate analysis were included in the multivariate analysis. All analyses were performed with SAS version 9.2 software (SAS Institute). A *P* of <.05 was considered to indicate statistical significance.

## 3 RESULTS

### 3.1 Patient characteristics

**FIGURE 2:**
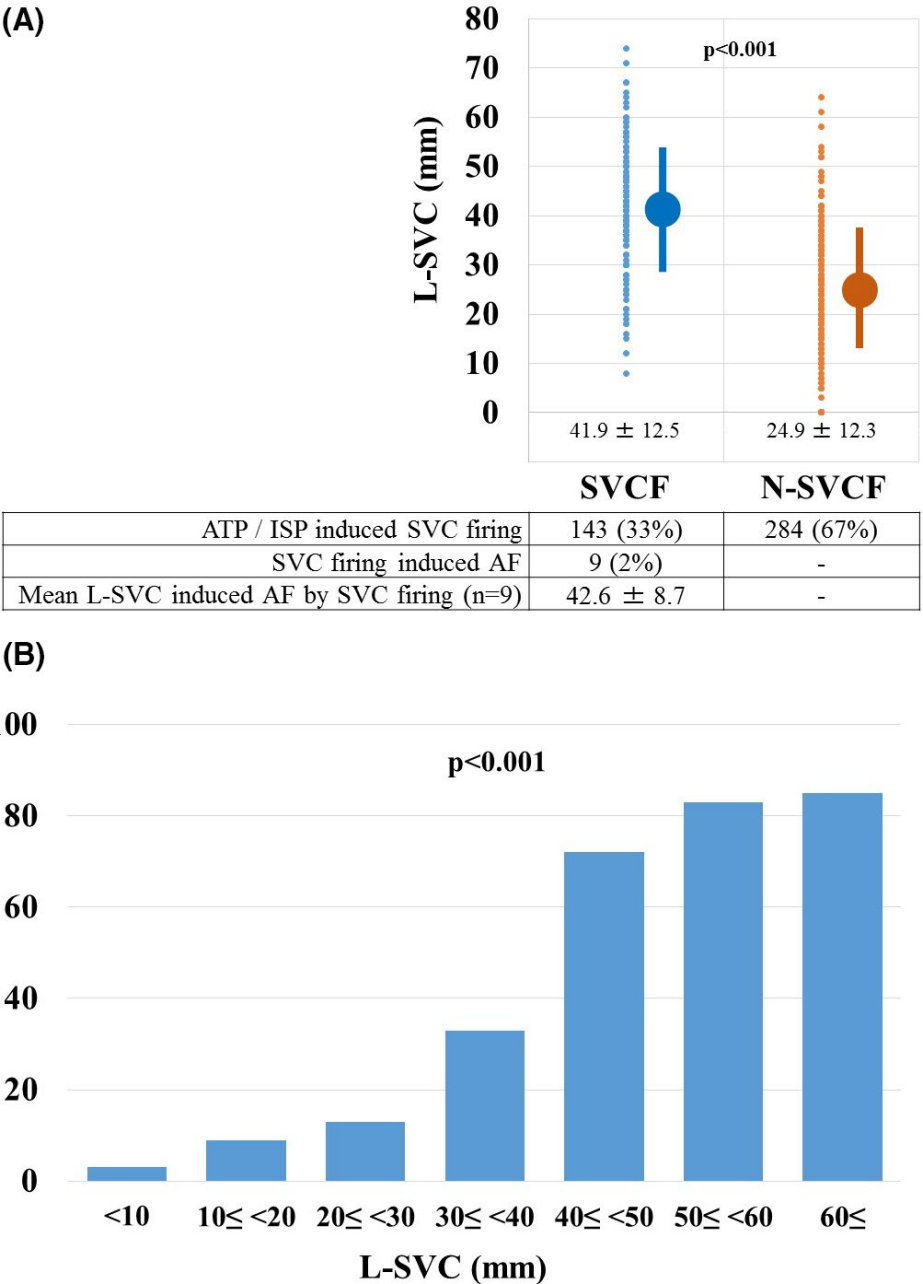
The length of superior vena cava (L‐SVC) was determined by the length from the top of the sinus node to the top of the myocardial sleeve in the SVC. The plotting of the L‐SVC, which was determined by the length from the top of the sinus node to the top of the myocardial sleeve in the SVC in the SVCF and N‐SVCF groups (A). The frequency of SVC firing according to the 7 L‐SVC groups (B). AF, atrial fibrillation; ATP, adenosine triphosphate; ISP, isoproterenol

Out of 427 patients, in 143 (33%) and 284 (67%) patients, SVC firing was induced (SVC firing [SVCF] group) and not induced (non‐SVC firing [N‐SVCF] group), respectively. The prevalence of radiofrequency ablation (80% vs 90%; *P* = .003), an L‐SVC (41.9 ± 12.5 mm vs 24.9 ± 12.3 mm; *P* < .001), longer (20.1 ± 2.7 mm vs 18.6 ± 4.3 mm; *P* < .001) and shorter diameters (18.6 ± 2.8 mm vs 17.3 ± 5.0 mm; *P* = .004) of SVC, and LAT (81.5 ± 22.7 points vs 75.8 ± 28.2 points; *P* = .035) were significantly lower and longer in the SVCF group than N‐SVCF group (Table 1; Figure 2A). Furthermore, out of 143 patients in the SVCF group, AF was induced by SVC firing in only nine patients (2%). The L‐SVC in those nine patients was 42.6 ± 8.7 mm.

**TABLE 1:** Patient characteristics

	All (n = 427)	SVCF (n = 143)	N‐SVCF (n = 284)	*P* value (SVCF vs N‐SVCF)
Male	281 (66%)	101 (71%)	180 (63%)	.137
Age (y)	71.6 ± 9.3	70.6 ± 9.6	72.2 ± 9.2	.099
Body mass index (kg/m^2^)	23.8 ± 9.8	23.7 ± 3.6	23.9 ± 11.7	.799
Body surface area (m^2^)	1.67 ± 0.19	1.68 ± 0.20	1.66 ± 0.19	.390
CHADS_2_ score	2.51 ± 1.26	2.42 ± 1.21	2.56 ± 1.29	.292
Type of atrial fibrillation				
Paroxysmal	212 (50%)	69 (48%)	143 (50%)	.695
Persistent	161 (38%)	52 (36%)	109 (38%)	—
Long‐lasting	54 (13%)	22 (15%)	32 (11%)	—
Underling heart disease				
Post‐percutaneous coronary intervention	36 (8%)	11 (8%)	25 (9%)	.698
Cardiomyopathy or amyloidosis	14 (3%)	4 (3%)	10 (4%)	.693
Post‐cardiac surgery	2 (1%)	0 (0%)	2 (1%)	.316
Post‐pacemaker implantation	16 (4%)	5 (3%)	11 (4%)	.847
Others	2 (1%)	1 (0.7%)	1 (0.4%)	.621
Laboratory analysis				
Brain natriuretic peptide (pg/mL)	165 ± 275	152 ± 171	172 ± 314	.485
Serum creatinine (mg/dL)	0.92 ± 0.24	0.89 ± 0.23	0.93 ± 0.24	.098
Left ventricular ejection fraction (%)	63.0 ± 10.4	62.7 ± 11.3	63.3 ± 9.9	.597
Diameter of left atrium (mm)	40.7 ± 6.8	40.8 ± 11.3	40.6 ± 6.9	.835
EPS/RFCA before the SVC evaluation				
Pulmonary vein antrum isolation	427 (100%)	143 (100%)	284 (100%)	1.000
Left atrial posterior (Box) isolation	188 (44%)	67 (47%)	121 (43%)	.405
Cavo‐tricuspid isthmus line ablation	102 (24%)	36 (25%)	66 (23%)	.659
Others	16 (4%)	4 (3%)	12 (4%)	.464
Radio‐frequency ablation	372 (87%)	115 (80%)	257 (90%)	.003
Cryoballoon ablation	55 (13%)	28 (20%)	27 (10%)	—
SVC evaluation				
Three‐dimensional mapping points by EnSite	200 ± 14	199 ± 14	200 ± 14	.565
L‐SVC (mm)	30.6 ± 14.7	41.9 ± 12.5	24.9 ± 12.3	<.001
Longer diameter of SVC (mm)	19.1 ± 3.9	20.1 ± 2.7	18.6 ± 4.3	<.001
Shorter diameter of SVC (mm)	17.7 ± 4.4	18.6 ± 2.8	17.3 ± 5.0	.004
Local activation time (msec)	77.7 ± 26.6	81.5 ± 22.7	75.8 ± 28.2	.035
Medications on admission				
Oral anticoagulation with NOAC or VKA	427 (100%)	143 (100%)	284 (100%)	1.000
Bepridil	339 (79%)	113 (79%)	226 (80%)	.894
Amiodarone	28 (7%)	10 (7%)	18 (6%)	.797
Class I agent	95 (22%)	36 (25%)	59 (21%)	.303
Digitalis	8 (2%)	4 (3%)	4 (1%)	.319
Beta‐blocker	335 (78%)	111 (78%)	224 (79%)	.767
ACE inhibitors or angiotensin II receptor blocker	288 (67%)	94 (66%)	194 (68%)	.593
Mineralocorticoid‐receptor antagonist	149 (35%)	46 (32%)	103 (36%)	.403
Diuretic	169 (40%)	61 (43%)	108 (38%)	.357
Statin	210 (49%)	62 (43%)	148 (52%)	.088
Platelet inhibitor	62 (15%)	20 (14%)	42 (15%)	.825

Abbreviations: ACE, angiotensin‐converting enzyme; EPS, electrophysiological study; L‐SVC, Length from top of sinus node to top of myocardial sleeve in the SVC; NOAC, non‐vitamin K antagonist oral anticoagulant; N‐SVCF, the non‐SVC firing group; RFCA, radiofrequency catheter ablation; SVC, superior vena‐cava; SVCF, the SVC firing group; VKA, vitamin K antagonist.

### 3.2 L‐SVC for the different types of AF

The L‐SVCs for all types of AF were significantly longer in the SVCF group than N‐SVCF group (all; *P* < .001) (Table 2). In the SVCF group, the L‐SVC in long‐lasting AF tended to be longer than that of paroxysmal and persistent AF, but was not significant. Furthermore, in the N‐SVCF group, the L‐SVC in long‐lasting AF tended to be shorter than that in paroxysmal and persistent AF, but it was not significant.

**TABLE 2:** L‐SVC for the different types of atrial fibrillation

	All	SVCF	N‐SVCF	*P* value
Paroxysmal	31.6 ± 14.1 (n = 212)	41.7 ± 13.4 (n = 69)	26.7 ± 11.7 (n = 143)	<.001
Persistent	29.7 ± 15.2 (n = 161)	41.4 ± 12.5 (n = 52)	24.1 ± 13.1 (n = 109)	<.001
Long‐lasting	29.9 ± 15.7 (n = 54)	44.1 ± 9.5 (n = 22)	20.1 ± 10.8 (n = 32)	<.001
*P* value	.893	.667	.094	—

Abbreviations: L‐SVC, length from the top of the sinus node to the top of the myocardial sleeve in the SVC; N‐SVCF, the non‐SVC firing group; SVCF, the SVC firing group.

### 3.3 SVC firing and the L‐SVC

**FIGURE 3:**
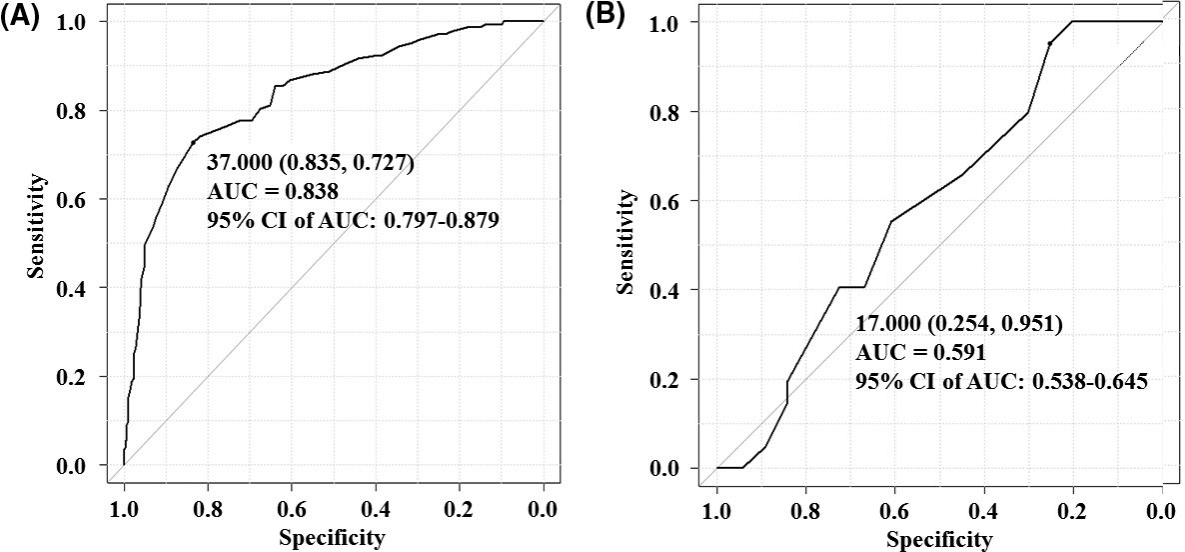
The length of superior vena cava (SVC) (L‐SVC) (A) was determined by the length from the top of the sinus node to the top of the myocardial sleeve in the SVC by EnSite™ images. The longer diameter of superior vena cava SVC (B) was determined by the diameter of SVC of the 1 cm above of junction of right atrium and SVC by computed tomography for radiofrequency of catheter ablation of atrial fibrillation. A receiver‐operating characteristic curve analysis of the L‐SVC (A) and longer diameter of SVC (B). The specificity and sensitivity of the SVC firing according to the L‐SVC and longer diameter of SVC were 0.835 and 0.727, for an L‐SVC of 37.00 mm, and 0.254 and 0.951, for longer diameter of SVC of 17.00 mm, respectively

The L‐SVC was significantly longer in the SVCF group than N‐SVCF group (41.9 ± 12.5 points vs 24.9 ± 12.3 points; *P* < .001). Therefore, the patients were divided into seven groups according to the L‐SVC. In accordance with the L‐SVC, the frequency of SVC firing significantly increased (*P* < .001) (Figure 2B). The ROC curve analysis of L‐SVC (Figure 3A) and longer diameter of SVC (Figure 3B) revealed that the specificity and sensitivity of SVC firing were 0.835 and 0.727, and 0.254 and 0.951, respectively, for a 37.0 mm L‐SVC and a 17.0 mm longer diameter of SVC. The areas under the curve (AUC) and 95% CI of the AUC were 0.838 and 0.797‐0.879, and 0.591 and 0.538‐0.645, respectively.

### 3.4 Independent risk factors of SVC firing in patients with AF who underwent RFCA of AF

A univariate analysis and multivariate statistical analysis revealed that the L‐SVC longer than 37.0 mm (odds ratio 6.39) and longer diameter of SVC longer than 17.0 mm (odds ratio 6.78) were independent risk factors for SVC firing in patients with AF who underwent RFCA of AF (Table 3). In view of these findings, L‐SVC longer than 37.0 mm and longer diameter of SVC longer than 17.0 mm may be one of the important predictors of SVC firing in patients with AF.

**TABLE 3:** Univariate and multivariate analyses

	Univariate analysis		Multivariate analysis	
	OR (95% CI)	*P* value	OR (95% CI)	*P* value
Male	1.39 (0.88‐2.20)	.160		
Age (y)	0.98 (0.96‐1.00)	.100		
Body mass index (kg/m^2^)	0.99 (0.97‐1.02)	.801		
Body surface area (m^2^)	1.59 (0.55‐4.60)	.389		
CHADS_2_ score	0.92 (0.78‐1.08)	.291		
Type of atrial fibrillation				
Paroxysmal	0.92 (0.60‐1.40)	.758		
Persistent	0.92 (0.59‐1.42)	.751		
Long‐lasting	1.43 (0.75‐2.66)	.280		
Underling heart disease				
Post‐percutaneous coronary intervention	0.86 (0.37‐1.88)	.854		
Cardiomyopathy or amyloidosis	0.78 (0.17‐2.79)	.782		
Post‐cardiac surgery	0.00 (0‐10.58)	.553		
Post‐pacemaker implantation	0.89 (0.24‐2.87)	1.000		
Others	1.98 (0.02‐156.79)	1.000		
Laboratory analysis				
Brain natriuretic peptide (pg/mL)	1.00 (0.99‐1.00)	.491		
Serum creatinine (mg/dL)	0.48 (0.19‐1.15)	.098		
Left ventricular ejection fraction (%)	0.99 (0.97‐1.01)	.597		
Diameter of the left atrium (mm)	1.00 (0.97‐1.03)	.835		
EPS/RFCA before SVC evaluation				
Pulmonary vein antrum isolation	—	—		
Left atrial posterior (Box) isolation	1.18 (0.77‐1.81)	.411		
Cavo‐tricuspid isthmus line ablation	1.11 (0.67‐1.81)	.718		
Others	0.65 (0.15‐2.21)	.594		
SVC evaluation				
L‐SVC (≥37.0 mm)	13.3 (8.07‐22.47)	<.001	6.39 (3.67‐11.1)	<.001
Longer diameter of SVC (mm)	6.60 (2.95‐14.8)	<.001	6.78 (2.67‐17.20)	<.001
Shorter diameter of SVC (mm)	1.07 (1.02‐1.12)	.004	1.03 (0.96‐1.11)	.408
Local activation time (msec)	0.99 (0.98‐0.99)	.036	0.99 (0.98‐1.00)	.199
Medications on admission				
Oral anticoagulation with NOAC or VKA	—	—		
Bepridil	0.96 (0.57‐1.64)	.900		
Amiodarone	1.11 (0.44‐2.62)	.837		
Class I agent	1.28 (0.77‐2.11)	.325		
Digitalis	2.01 (0.37‐10.96)	.450		
Beta‐blocker	0.93 (0.55‐1.56)	.803		
ACE inhibitors or angiotensin II receptor blocker	0.89 (0.56‐1.39)	.587		
Mineralocorticoid‐receptor antagonist	0.83 (0.53‐1.30)	.452		
Diuretic	1.21 (0.78‐1.86)	.402		
Statin	0.70 (0.45‐1.07)	.101		
Platelet inhibitor	0.94 (0.49‐1.71)	.885		

Abbreviations: ACE, angiotensin‐converting enzyme; EPS, electrophysiological study; NOAC, non‐vitamin K antagonist oral anticoagulant; RFCA, radiofrequency catheter ablation; SVC, Length from top of sinus node to top of myocardial sleeve in the SVC; VC, superior vena‐cava; VKA, vitamin K antagonist.

### 3.5 SVC isolation

Out of 143 patients with SVC firing, in 119 (83%), a complete SVC isolation was additionally performed. RFCA of the SVC isolation lines was performed about 10 mm above the top of the sinus node which was detected by EnSite™. However, in the remaining 24 patients, a complete SVC isolation could not be achieved because of capture of the phrenic nerve by 10 V pacing from the ablation catheter on the SVC isolation lines to avoid the phrenic nerve palsy. Fortunately, in nine patients with SVC firing, which induced AF during an ISP and ATP infusion, a complete SVC isolation was achieved.

### 3.6 Complications

Out of 427 patients, in 12 (2.8%), the complications associated with procedure, including 5 (1.2%) cardiac tamponade, 2 (0.5%) pericarditis, 2 (0.5%) retroperitoneal hematoma, 1 (0.2%) acute paralytic gastric dilatation, 1 (0.2%) transient ischemic attack/brain infarction, and 1 (0.2%) transient diaphragmatic paralysis, were observed. There were no statistically differences in the complications between the SVCF and N‐SVCF groups (data not shown).

### 3.7 Recurrence of AF

After conclusion of the study, 14%, 21%, and 38% of the patients with paroxysmal, persistent, and long‐lasting AF had recurrence of AF, respectively, at the final follow up at 12 months post‐ablation.

## 4 DISCUSSION

### 4.1 The new findings and important points of this manuscript

The SVC is one of the most important sources of non‐PV foci revealed by the results of an ISP and ATP test, which can aid in the identification of an arrhythmogenic SVC during the RFCA procedure. It has been reported that the SVC, which harbors 25%‐40% of non‐PV foci, is the most common non‐PV origin of AF.^4^, ^9^, ^10^, ^11^ However, no reports have covered the relationship between the SVC firing during ISP and ATP, and the length of the myocardial sleeve in the SVC and diameter of SVC. We evaluated that and confirmed new findings and important points in the evaluation after the PVAI as follows: (a) Out of 427 patients who underwent RFCA of AF, in 143 (33%) SVC firing was induced by an intravenous administration of ISP and ATP (Figure 1D). (b) Out of 143 patients in whom SVC firing was induced, in only nine (6%) patients AF was induced by SVC firing (Figure 1E). (c) The L‐SVC (41.9 ± 12.5 mm vs 24.9 ± 12.3 mm; *P* < .001) and longer diameter of SVC (20.1 ± 2.7 mm vs 18.6 ± 4.3 mm; *P* < .001) were significantly longer in the SVCF group than N‐SVCF group (Table 1; Figure 2A). (4) In accordance with the L‐SVC, the frequency of SVC firing significantly increased (*P* < .001) (Figure 2B). (5) The ROC curve analysis (Figure 3A,B), univariate analysis, and multivariate statistical analysis (Table 3) revealed that the L‐SVC longer than 37.0 mm (odds ratio 6.39) and longer diameter of SVC longer than 17.0 mm were independent risk factors for SVC firing in patients with AF who underwent RFCA of AF.

### 4.2 LAT of SVC

The L‐SVC, and longer and shorter diameter of SVC were significantly longer in the SVCF group than the N‐SVCF group. Because, probably, longer and wider myocardial sleeve in the SVCF group may have much atrial muscle, LAT was longer than N‐SVCF group. However, longer LAT was not independent risk factors for SVC firing in patients with AF who underwent RFCA of AF in this study.

### 4.3 L‐SVC for the different types of AF

There were no statistically differences of L‐SVCs between the all types of AF including paroxysmal, persistent, and long‐lasting AF (Table 2).

### 4.4 Was the SVC isolation needed?

This study revealed that an L‐SVC longer than 37.0 mm and longer diameter of SVC longer than 17.0 mm may be one of the important predictors of SVC firing and/or greater arrhythmogenicity in patients with AF. Probably, a longer and wider myocardial sleeve and may include much atrial muscle, and with much atrial muscle, electrical firing may occur, which may initiate AF. In this study, in 143 (33%) patients SVC firing was induced by the intravenous administration of ISP and ATP (Figure 1D). However, out of 143 patients in whom SVC firing was induced, AF was induced by SVC firing in only nine (6%) patients (Figure 1E). In those nine patients, the SVC played an important role in initiating and maintaining AF. On the other hand, in the remaining 124 patients, AF could not be maintained AF because of the PVAI procedure, which isolated the most important area to maintain AF, even though triggered SVC firing occurred. Furthermore, in paroxysmal AF patients, empirically adding an SVCI after the PVAI did not significantly reduce the AF recurrence after the RFCA of AF.^9^ Possibly, in those patients, an additional SVC isolation may not be necessary after a complete PVAI. On the other hand, it has been shown that excessive atrial ectopy and short atrial runs increase the risk of the incidence of AF and strokes.^18^ In view of this point, the 124 patients with SVC firing without AF may undergo an SVC isolation. Whether an SVC isolation should be performed or not, should be determined in a randomized clinical trial and further studies.

### 4.5 Limitations of the study

This study had a prospective design but was not a randomized clinical trial. Although our study was a multi‐center trial, it was limited by the relatively small number of patients. The SVC mapping was performed after PVAI procedure. Because of the short follow‐up period, our study still could not demonstrate the long‐term clinical benefits. Whether our results can safely be extrapolated to the inclusion of a larger number of patients, and a longer follow‐up period for these patients by a randomized clinical trial should be determined in further studies.

## 5 CONCLUSIONS

In view of these findings, an L‐SVC longer than 37.0 mm (odds ratio 6.39) and longer diameter of SVC longer than 17.0 mm (odds ratio 6.78) may be one of the important predictors of SVC firing in patients with AF.

## CONFLICT OF INTEREST

The authors report no relationships that could be construed as a conflict of interest.

## AUTHORS CONTRIBUTIONS

All doctors were in charge of the patients in this study. Drs. Nyuta E and Takemoto M wrote this manuscript. Drs. Masumoto A and Takemoto M performed the radiofrequency catheter ablation of the patients in this study. Drs. Mito T and Takemoto performed the data analysis.

## References

[1] Haissaguerre M , Jais P , Shah DC , Takahashi A , Hocini M , Quiniou G , et al. Spontaneous initiation of atrial fibrillation by ectopic beats originating in the pulmonary veins. N Engl J Med. 1998;339:659–66. 9725923 10.1056/NEJM199809033391003

[2] Luik A , Radzewitz A , Kieser M , Walter M , Bramlage P , Hormann P , et al. Cryoballoon versus open irrigated radiofrequency ablation in patients with paroxysmal atrial fibrillation: the prospective, randomized, controlled, noninferiority freezeaf study. Circulation. 2015;132:1311–9. 26283655 10.1161/CIRCULATIONAHA.115.016871 PMC4590523

[3] Takahashi A , Iesaka Y , Takahashi Y , Takahashi R , Kobayashi K , Takagi K , et al. Electrical connections between pulmonary veins: implication for ostial ablation of pulmonary veins in patients with paroxysmal atrial fibrillation. Circulation. 2002;105:2998–3003. 12081994 10.1161/01.cir.0000019585.91146.ab

[4] Lin WS , Tai CT , Hsieh MH , Tsai CF , Lin YK , Tsao HM , et al. Catheter ablation of paroxysmal atrial fibrillation initiated by non‐pulmonary vein ectopy. Circulation. 2003;107:3176–83. 12821558 10.1161/01.CIR.0000074206.52056.2D

[5] Shah D , Haissaguerre M , Jais P , Hocini M . Nonpulmonary vein foci: do they exist? Pacing Clin Electrophysiol. 2003;26:1631–5. 12914614 10.1046/j.1460-9592.2003.t01-1-00243.x

[6] Di Biase L , Burkhardt JD , Mohanty P , Mohanty S , Sanchez JE , Trivedi C , et al. Left atrial appendage isolation in patients with longstanding persistent AF undergoing catheter ablation: belief trial. J Am Coll Cardiol. 2016;68:1929–40. 27788847 10.1016/j.jacc.2016.07.770

[7] Heeger CH , Rillig A , Geisler D , Wohlmuth P , Fink T , Mathew S , et al. Left atrial appendage isolation in patients not responding to pulmonary vein isolation. Circulation. 2019;139:712–5. 30689416 10.1161/CIRCULATIONAHA.118.037451

[8] Tsai CF , Tai CT , Hsieh MH , Lin WS , Yu WC , Ueng KC , et al. Initiation of atrial fibrillation by ectopic beats originating from the superior vena cava: electrophysiological characteristics and results of radiofrequency ablation. Circulation. 2000;102:67–74. 10880417 10.1161/01.cir.102.1.67

[9] Wang XH , Liu X , Sun YM , Shi HF , Zhou L , Gu JN . Pulmonary vein isolation combined with superior vena cava isolation for atrial fibrillation ablation: a prospective randomized study. Europace. 2008;10:600–5. 18442966 10.1093/europace/eun077

[10] Kuroi A , Miyazaki S , Usui E , Ichihara N , Kanaji Y , Takagi T , et al. Adenosine‐provoked atrial fibrillation originating from non‐pulmonary vein foci: the clinical significance and outcome after catheter ablation. JACC Clin Electrophysiol. 2015;1:127–35. 29759355 10.1016/j.jacep.2015.02.020

[11] Higuchi K , Yamauchi Y , Hirao K , Sasaki T , Hachiya H , Sekiguchi Y , et al. Superior vena cava as initiator of atrial fibrillation: factors related to its arrhythmogenicity. Heart Rhythm. 2010;7:1186–91. 20470902 10.1016/j.hrthm.2010.05.017

[12] Kurita T , Nogami A , Japanese Circulation Society Foundation/Japanese Heart Rhythm Society Task F . 2018 JCS/JHRS guideline on non‐pharmacotherapy of cardiac arrhythmias. https://www.j‐circ.or.jp/cms/wp‐content/uploads/2018/07/JCS2018_kurita_nogami191120.pdf.2018

[13] Antoku Y , Takemoto M , Mito T , Masumoto A , Nozoe M , Tanaka A , et al. Evaluation of coronary artery disease in patients with atrial fibrillation by cardiac computed tomography for catheter ablation – a CADAF‐CT trial 2. Intern Med. 2020;59(22):2831–7. 32713911 10.2169/internalmedicine.4745-20 PMC7725621

[14] Hida S , Takemoto M , Masumoto A , Mito T , Nagaoka K , Kumeda H , et al. Clinical benefits of deep sedation with a supraglottic airway while monitoring the bispectral index during catheter ablation of atrial fibrillation. J Arrhythm. 2017;33:283–8. 28765758 10.1016/j.joa.2017.04.001 PMC5529590

[15] Mito T , Takemoto M , Antoku Y , Masumoto A , Nozoe M , Kinoshita S , et al. Evaluation of coronary artery disease in patients with atrial fibrillation by cardiac computed tomography for catheter ablation: CADAF‐CT trial. Heart Vessels. 2020;35:1037–43. 32140769 10.1007/s00380-020-01572-6 PMC7332475

[16] Theiler L , Gutzmann M , Kleine‐Brueggeney M , Urwyler N , Kaempfen B , Greif R . I‐gel supraglottic airway in clinical practice: a prospective observational multicentre study. Br J Anaesth. 2012;109:990–5. 22956643 10.1093/bja/aes309

[17] Yamaguchi T , Tsuchiya T , Miyamoto K , Nagamoto Y , Takahashi N . Characterization of non‐pulmonary vein foci with an ensite array in patients with paroxysmal atrial fibrillation. Europace. 2010;12:1698–706. 21097479 10.1093/europace/euq326

[18] Larsen BS , Kumarathurai P , Falkenberg J , Nielsen OW , Sajadieh A . Excessive atrial ectopy and short atrial runs increase the risk of stroke beyond incident atrial fibrillation. J Am Coll Cardiol. 2015;66:232–41. 26184616 10.1016/j.jacc.2015.05.018

